# Predictive Value of the Age, Creatinine, and Ejection Fraction (ACEF) Score in Patients With Acute Fulminant Myocarditis

**DOI:** 10.3389/fphys.2021.596548

**Published:** 2021-02-24

**Authors:** Lin Liu, Xinyu Yang, Yiyu Gu, Tingbo Jiang, Jialiang Xu, Mingzhu Xu

**Affiliations:** Department of Cardiology, The First Affiliated Hospital of Soochow University, Suzhou, China

**Keywords:** age, creatinine, left ventricular ejection fraction, risk prediction, fulminant myocarditis

## Abstract

**Objective:**

Patients with acute fulminant myocarditis often have more adverse cardiovascular events and higher mortality. The purpose of this study was to evaluate the usefulness of age, creatinine, and left ventricular ejection fraction (ACEF score), in determining the risk that acute fulminant myocarditis will lead to serious cardiovascular events, death, and cardiac dysfunction.

**Methods:**

We retrospectively reviewed the demographics, laboratory tests, medications, echocardiographic examinations, in-hospital clinical outcomes, major adverse cardiovascular events (MACE), and survival rate at 1 year in the medical records of 220 consecutive subjects suffering from acute fulminant myocarditis from January 2013 to June 2019.

**Results:**

Two hundred twenty patients were divided into a survivor group and a non-survivor group. This study found that patients in the non-survivor group were older, had higher heart rates, and had more serious injuries to multiple organ functions. A high ACEF score at admission was independently associated with an unfavorable prognosis, and it was a predictor of in-hospital mortality. The current analysis extends the predictive performance of the ACEF scores at 30 days by evaluating echocardiographic data as applied to survivors of fulminant myocarditis and cumulative rates of MACE at 1 year. The results indicated that patients with high ACEF scores had poor recovery of cardiac function, and higher rates of MACE, all-cause death, and heart failure at 1 year than the low-ACEF group.

**Conclusion:**

The ACEF score was identified as an effective predictor of poor in-hospital outcomes, worse cardiac recovery after 30 days, and higher rates of MACE, all-cause death, and heart failure at 1 year in patients who had acute fulminant myocarditis. These data suggest that its predictive accuracy means the ACEF score could be used to assess the prognosis of patients with acute fulminant myocarditis.

## Introduction

Acute myocarditis is an autoimmune inflammation of the myocardium to the possible sources with the expression of various clinical manifestations, myocardial damage, hemodynamic disorders, severe arrhythmias, and unfavorable prognosis (McCarthy et al., 2000; Eckart et al., 2004; Gupta et al., 2008; Sharma et al., 2019). Despite the considerably high risk of heart attack, life-threatening arrhythmias and shock, patients with acute fulminant myocarditis might recover and survive longer if they live through the acute phase and if their cardiac function recovers within 1 month (McCarthy et al., 2000; Ammirati et al., 2017; Sharma et al., 2019). Thus, early recognition and risk stratification would lower the in-hospital mortality in such patients if impressive advances in medical therapeutic measurements and aggressive mechanical circulatory support were used earlier (Diddle et al., 2015; Li et al., 2019).

A number of risk factors have been associated with in-hospital mortality and longer-term outcomes in patients who suffer from acute fulminant myocarditis, especially renal dysfunction and impaired cardiac function (Yang et al., 2012; Xu et al., 2018). However, until now, there have been few simple and effective tools to evaluate the in-hospital and 30 day prognosis and long-term survival in patients after acute fulminant myocarditis. The age, creatinine, and left ventricular ejection fraction (ACEF) score was originally developed to predict 1 year mortality in patients who survived for >30 days after acute myocardial infarction (Lee et al., 2015) and to assess mortality risk in elective cardiac operations (Ranucci et al., 2009). Its use has subsequently been extended to other clinical conditions, including acute coronary syndrome, infective endocarditis, and transcatheter aortic valve implantation (Di Serafino et al., 2014; Arai et al., 2015; Stähli et al., 2018; Wei et al., 2019). However, the prognostic value of the ACEF score in patients with acute fulminant myocarditis has not been evaluated. In line with this notion, this study aimed to determine whether the ACEF score is associated with mortality and to investigate the prognostic value of the ACEF score for patients with fulminant myocarditis. The results might help clinical physicians in clinical assessment and decision-making.

## Materials and Methods

### Study Population

This was a retrospective, single-center observational study of 225 patients diagnosed with fulminant myocarditis who were admitted to a cardiac intensive care unit between January 2013 and June 2019. The procedures of the study conformed to the Helsinki Declaration with regard to ethical principles, and use of the participants’ data was in accordance with the ethical standards of the institutional committees. All authors confirmed that each patient’s information was identified by an alias. The data were collected and divided into survivor and non-survivor groups. The patients standard transthoracic echocardiography at admission.

### Data Collection

Each patient’s clinical characteristics, clinical manifestations, laboratory examinations, echocardiographic data, and ACEF score were collected and analyzed. The clinical characteristics included gender, age, prior hypertension, prior diabetes mellitus, alcohol use, and smoking. The clinical manifestations referred to heart rate, mean arterial blood pressure, respiratory symptoms, alimentary symptoms, fever, chest tightness or dyspnea, chest pain, and neurological symptoms. Laboratory biomarkers, including white blood cell count (WBC counts, reference value 3.5–9.5 × 10E12/L), hemoglobin (reference value 115–160 g/L), MB isoenzyme of creatine kinase (CK-MB, reference value 0–24 U/L), total bilirubin (normal range 3.4–17.1 μmol/L), and serum creatinine (Scr, normal range 0.7–1.5 mg/dL), were measured at admission. Cardiac structure and function were evaluated based on echocardiographic changes in left atrium dimensions (LAd), left ventricular end systolic dimensions (LVESd), left ventricular end diastolic dimensions (LVEDd), left ventricular ejection fraction (LVEF), pericardial effusion, weakening motion of the ventricular wall, and valve regurgitation. These echocardiographic data were measured with M-mode and two-dimensional Doppler echocardiography. The ACEF score was calculated according to the following formula: ACEF = age/LVEF+1 (if creatinine was >2.0 mg/dL) (Ranucci et al., 2009).

The variables related to incidence of death in subjects were analyzed using multivariate logistic regression to identify independent predictors. All enrolled patients were then divided into two groups according to their ACEF score at admission: a low ACEF score group (ACEF score ≤ 1.43) and a high ACEF score group (ACEF score > 1.43). The clinical characteristics, laboratory examinations, and echocardiography at admission were examined according to different levels of ACEF scores. In addition, therapeutic treatments and strategies, as well as in-hospital complications [shock, New York Association (NYHA class), multiple organ failure, and death] between the group with low ACEF scores and the group with high ACEF scores were analyzed. The therapeutic treatments and strategies included intravenous injection of medication (vitamin C, immunoglobulin, methylprednisolone, diuretics, dopamine, norepinephrine, inotropic agents), oral administration of medication (renin-angiotensin system inhibitors, beta-receptor blockers, aldosterone antagonists), and other medical assistance such as temporary pacemaker, ventilator support, intra-aortic balloon pump (IABP), continuous renal replacement therapy (CRRT), and extracorporeal membrane oxygenation (ECMO). In addition, for survivors after 1 month, the echocardiographic data between the low-ACEF group and the high-ACEF group were compared. Patients with fulminant myocarditis were followed up for 1 year. Major adverse cardiovascular events (MACE) were defined as the composite of all-cause death, heart failure, and readmission. The 1 year all-cause death and the data of clinical follow-up were obtained by reviewing medical records and through telephone interviews with patients on.

### Statistical Analysis

Statistical analysis was performed using the SPSS software package (version 19.0, SPSS, United States). Continuous variables were expressed as mean ± standard deviation when normally distributed, and they were compared using the independent-sample *t*-test or Mann Whitney *U*-test. Otherwise, comparison was made using the Wilcoxon test and shown as median (quartile range). Categorical variables were presented as numbers (percentages), and they were compared with Pearson’s chi-square test or Fisher’s exact test. Multivariate logistic regression was performed to determine independent predictors of in-hospital death in the subjects. The accuracy of the ACEF score in predicting mortality was assessed using receiver operating characteristic (ROC) curve analysis. Through ROC curve analysis, the optimum cut-off ACEF value was determined as the point of the highest Youden index (sensitivity + specificity − 1). Patients were categorized into two groups according to the statistical ACEF score: the low-ACEF group and the high-ACEF group. The 1 year rates of cumulative MACE events were evaluated by the Kaplan-Meier method, and the difference between groups was assessed by log-rank test in patients with acute fulminant myocarditis. A *p* < 0.05 (two-sided) was defined as statistically significant.

## Results

### Patients’ Clinical Characteristics, Performance, Laboratory Findings, Echocardiographic Examination, and ACEF Scores

The 225 patients with fulminant myocarditis were enrolled, and 5 patients were excluded because of incomplete data. Among the remaining 220 patients, 24 (10.91%) died in hospital and were classified as a non-survivor group. The other 196 patients were classified into a survivor group. The baseline characteristics, clinical manifestations, laboratory data, echocardiographic measurements, and ACEF scores at admission are presented in Table 1. Differences between the two groups in gender, proportion of prior medical histories, mean arterial blood pressure, frequency of clinical presentation, and hemoglobin level did not reach statistical significance. With respect to echocardiographic data (LAd, LVEDd, pericardial effusion, weakening motion of the ventricular wall, and valve regurgitation), patients who suffered acute fulminant myocarditis in the non-survivor group had no significant difference when compared with patients in the survivor group (Table 1).

**TABLE 1:** Comparison of the clinical features and the ACEF score in patients with acute fulminant myocarditis.

Variables	Survivor	Non-survivor	*P*-value
	(*n* = 196)	(*n* = 24)	
**Clinical characteristics**			
Gender (male) [*n* (%)]	123 (62.76%)	15 (62.5%)	0.981
Age (years)	35.00 (24.25∼49.75)	52.63 ± 18.08*	0.001
Prior hypertension [*n* (%)]	32 (16.33%)	6 (25.00%)	0.267
Prior diabetes mellitus [*n* (%)]	13 (6.63%)	2 (8.33%)	0.671
Alcohol [*n* (%)]	22 (11.22%)	3 (12.50)	0.741
Smoking [*n* (%)]	44 (22.45%)	4 (16.67%)	0.517
Heart rate (bpm)	80.67 ± 23.76	115.58 ± 28.90*	0.000
Mean arterial blood pressure (mmHg)	80.48 ± 13.33	79.28 ± 23.70	0.809
**Clinical manifestation**			
Respiratory symptom [*n* (%)]	63 (32.14%)	11 (32.14%)	0.180
Alimentary symptom [*n* (%)]	47 (23.98%)	9 (37.5%)	0.151
Fever *n* [*n* (%)]	111 (56.63%)	17 (70.83%)	0.183
Chest tightness or dyspnea [*n* (%)]	137 (69.90%)	20 (83.33%)	0.169
Chest pain [*n* (%)]	54 (27.55%)	5 (20.83%)	0.483
Neurological symptom (syncope) [*n* (%)]	36 (18.37%)	7 (29.17%)	0.161
**Laboratory examination**			
White blood cell counts (×10 E12/L)	8.61 (6.10∼11.89)	13.77 ± 8.82*	0.041
Hemoglobin (g/L)	131.51 ± 20.65	137.17 ± 27.47	0.348
CK-MB (U/L)	25.61 (9.21∼63.84)	95.37 ± 66.45*	0.000
Total bilirubin (μmol/L)	12.70 (9.20∼17.80)	25.26 ± 20.46*	0.028
Serum creatinine (mg/dL)	0.83 (0.65∼1.05)	1.66 (0.95∼1.91)*	0.000
**Echocardiographic parameters**			
LAd (mm)	35.85 ± 6.00	37.86 ± 7.59	0.148
LVESd (mm)	36.00 (32.00∼40.00)	40.29 ± 6.81*	0.002
LVEDd (mm)	49.0 (46.00∼53.00)	49.36 ± 5.31	0.961
LVEF	0.51 (0.40∼0.61)	0.34 ± 0.08*	0.000
Pericardial effusion [*n* (%)]	66 (33.67%)	11 (45.83%)	0.238
Weakening motion of the ventricular wall [*n* (%)]	108 (55.10%)	17 (70.83%)	0.142
Valve regurgitation [*n* (%)]	72 (36.73%)	14 (58.33%)	0.105
ACEF score	0.74 (0.49∼1.15)	2.14 ± 0.94*	0.000

Values are given as mean ± standard deviation, median and interquartile range or number and percentages. *P < 0.05 (survivor group vs. non-survivor group). CK-MB, MB isoenzyme of creatine kinase; LAd, left atrium diameter; LVESd, left ventricular end-systolic diameter; LVEDd, left ventricular end-diastolic diameter; LVEF, left ventricular ejection fraction; ACEF score, the age, creatinine, and ejection fraction score.

The patients with fulminant myocarditis in the non-survivor group were older [52.63 ± 18.08 vs. 35.00 (24.25∼49.75)], and they had higher heart rates (115.58 ± 28.90 vs. 80.67 ± 23.76 bpm) than the survivors who complicated acute fulminant myocarditis. Patients who did not survive after fulminant myocarditis had higher WBC counts [13.77 ± 8.82 vs. 8.61 (6.10∼11.89) × 10E12/L), CK-MB [95.37 ± 66.45 vs. 25.61 (9.21∼63.84) U/L], total bilirubin [25.26 ± 20.46 vs. 12.70 (9.20∼17.80) μmol/L), and serum creatinine [1.66 (0.95∼1.91) vs. 0.83 (0.65∼1.05) mg/dL] at admission compared to survivors. In addition, patients with acute fulminant myocarditis who did not survive had a significantly higher mean LVESd [40.29 ± 6.81 vs. 36.00 (32.00∼40.00) mm], and a dramatically lower LVEF [0.34 ± 0.08 vs. 0.51 (0.40∼0.61)] in comparison with the patients who survived. Importantly, ACEF scores were higher in patients in the non-survivor group than in the survivor group (Table 1).

### Clinical Outcomes and Predictors of In-Hospital Death

**FIGURE 1:**
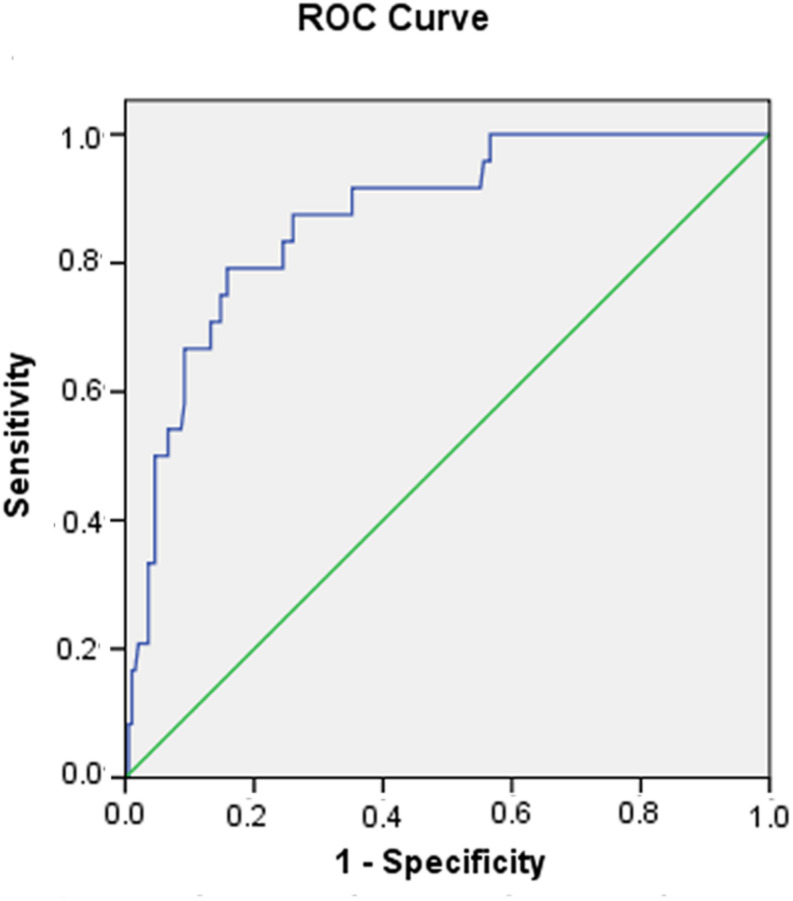
Receiver operating characteristic (ROC) curve of the ACEF score in predicting in-hospital death in patients with acute fulminant myocarditis.

Six risk factors (heart rate, WBC count, CK-MB, total bilirubin, LVESd, and ACEF) were ranked for predicting in-hospital death. Multivariate logistic regression demonstrated that the ACEF score [odds ratio (OR): 4.499; 95% confidence interval (CI): (0.960–1.061); *p* < 0.000] was confirmed to be a strong independent predictor of in-hospital death in patients with acute fulminant myocarditis in contrast to other risk factors (Table 2). The ACEF score displayed good prognostic information for in-hospital mortality based on ROC curve analysis, and the area of ROC was 0.871 (Figure 1).

**TABLE 2:** The predictors of in-hospital mortality in patients with acute fulminant myocarditis by multivariate logistic regression analysis.

Variables	Odds ratio (95% CI)	*P-*value
Heart rate (bpm)	1.028 (0.997–1.060)	0.081
White blood cell counts (×10 E12/L)	1.019 (0.930–1.118)	0.685
Primary CK-MB (U/L)^*a*^	1.006 (0.998–1.015)	0.159
Primary total bilirubin	1.009 (0.960–1.061)	0.718
Left ventricular end-systolic dimension (mm)	0.982 (0.892–1.080)	0.704
ACEF score^*b*^	4.499 (0.960–1.061)	0.000*

*P < 0.05. ^*a*^CK-MB, MB isoenzyme of creatine kinase. ^*b*^ACEF score, the age, creatinine, and ejection fraction score.

### Evaluation of Clinical Characteristics, Laboratory Tests, Echocardiographic Findings on Admission, In-Hospital Medical Treatments, and Clinical Complications

A recent study reported on the relationship between ACEF scores and all-cause mortality in patients with acute coronary syndrome (Stähli et al., 2018). Based on ROC curve analysis, it was determined that an ACEF score of 1.43 was the optimum cut-off value, since it had the highest Youden index. Therefore, the patients were reclassified into two groups according to their ACEF scores. A low ACEF score (≤1.43, *n* = 170) indicated a low risk of death, and a high ACEF score (>1.43, *n* = 50) indicated a high risk of death.

Gender, frequency of alcohol use, and frequency of smoking had no significant difference between the low-ACEF group and the high-ACEF group. The patients in the high-ACEF group were older, and more of them had a history of hypertension and diabetes. This indicated that older patients or patients with more clinical diseases might have a higher risk of death.

The differences between the low and high ACEF groups in echocardiographic measurements on admission were analyzed. There was no statistically significant difference between the two groups with regard to pericardial effusion, weakening ventricular wall motion, and valve regurgitation. By contrast, patients with fulminant myocarditis in the high-ACEF group had higher LAd (39.51 ± 6.65 vs. 35.05 ± 5.68 mm, *p* < 0.05), LVESd [41.40 ± 6.22 vs. 34.00 (31.00∼39.00) mm, *p* < 0.05), and LVEDd [50.94 ± 5.30 vs. 48.00 (46.00∼51.00) mm, *p* < 0.05], but a notable decrease in LVEF [0.37 ± 0.09 vs. 0.55 (0.42∼0.62), *p* < 0.05) than the low-ACEF group (Table 3). These results demonstrated that patients with high ACEF scores had more serious cardiac dysfunction than the patients with low ACEF scores (Table 3).

**TABLE 3:** Summary of the clinical features according to ACEF score in patients with acute fulminant myocarditis.

	ACEF score ≤ 1.43 (*n* = 170)	ACEF score > 1.43 (*n* = 50)	*P*-value
**Clinical characteristics**			
Gender (male) [*n* (%)]	107 (62.94%)	31 (62.00%)	0.904
Age (years)	34.64±14.34	59.34±15.64*	0.000
Prior hypertension [*n* (%)]	16 (9.41%)	22 (44.00%)*	0.000
Prior diabetes mellitus [*n* (%)]	7 (4.12%)	8 (16.00%)*	0.009
Alcohol [*n* (%)]	18 (10.59%)	7 (14.00%)	0.473
Smoking [*n* (%)]	35 (20.59%)	13 (26%)	0.415
**Laboratory examination**			
White blood cell counts (×10 E12/L)	8.12 (5.97∼11.16)	13.62 ± 8.19*	0.000
Hemoglobin (g/L)	132.08 ± 21.66	132.20 ± 20.99	0.973
CK-MB (U/L)	24.92 (8.69∼56.69)	75.64 ± 59.00*	0.001
Total bilirubin (μmol/L)	13.77 ± 7.27	15.80 (10.70∼28.70)*	0.001
Serum creatinine (mg/dL)	0.82 ± 0.24	1.75 (1.29∼2.30)*	0.000
**Echocardiographic data on admission**	***n* = 170**	***n* = 50**	
LAd (mm)	35.05 ± 5.68	39.51 ± 6.65*	0.000
LVESd (mm)	34.00 (31.00∼39.00)	41.40 ± 6.22*	0.000
LVEDd (mm)	48.00 (46.00∼51.00)	50.94 ± 5.30*	0.005
LVEF	0.55 (0.42∼0.62)	0.37 ± 0.09*	0.000
Pericardial effusion [*n* (%)]	52 (30.59%)	14 (28.00%)	0.726
Weakening motion of ventricular wall [*n* (%)]	76 (44.71%)	22 (44.00%)	0.930
Valve regurgitation [*n* (%)]	50 (29.41%)	22 (44.00%)	0.053
**Medical treatments**	**(*n* = 170)**	**(*n* = 50)**	
Renin-angiotensin system inhibitor [*n* (%)]	81 (47.65%)	22 (44.00%)	0.650
Beta receptor blocker [*n* (%)]	89 (52.35%)	19 (38.00%)	0.074
Aldosterone antagonist [*n* (%)]	59 (34.71%)	18 (36.00%)	0.866
Vitamin C [*n* (%)]	153 (90.00%)	44 (88.00%)	0.000
Immunoglobulin [*n* (%)]	101 (59.41%)	34 (68.00%)	0.273
Methylprednisolone [*n* (%)]	123 (72.35%)	38 (76.00%)	0.609
Diuretics [*n* (%)]	60 (35.29%)	38 (76.00%)*	0.000
Dopamine [*n* (%)]	30 (17.65%)	25 (50.00%)*	0.000
Norepinephrine [*n* (%)]	19 (11.18%)	23 (46.00%)*	0.000
Inotropic agent [*n* (%)]	8 (4.71%)	21 (42.00%)*	0.000
Temporary pacemaker [*n* (%)]	32 (18.82%)	8 (16.00%)	0.649
Ventilator support [*n* (%)]	19 (11.18%)	26 (52.00%)*	0.000
Intra-aortic balloon pump [*n* (%)]	14 (8.24%)	20 (40.00%)*	0.000
CRRT [*n* (%)]	2 (1.18%)	17 (34.00%)*	0.000
ECMO [*n* (%)]	1 (1.43%)	4 (8.00%)*	0.002
**Clinical complication**			
Shock [*n* (%)]	35 (20.59%)	34 (68.00%)*	0.000
**NYHA**			
Grade I-II [*n* (%)]	112 (65.88%)	17 (34.00%)*	0.000
Grade III-IV [*n* (%)]	58 (34.12%)	33 (66.00%)*	0.000
VT/VF [*n* (%)]	14 (8.24%)	19 (38.00%)*	0.000
Multiple organ failure [*n* (%)]	47 (27.65%)	38 (76.00%)*	0.000
Death [*n* (%)]	5 (2.94%)	19 (38.00%)*	0.000

Values are presented as mean ± standard deviation, median and interquartile range or number and percentages. *P < 0.05 (ACEF score > 1.43 vs. ACEF score ≤ 1.43). ACEF, the age, creatinine, and left ventricular ejection fraction; CK-MB, MB isoenzyme of creatine kinase; Lad, left atrium diameter; LVESd, left ventricular end-systolic diameter; LVEDd, left ventricular end-diastolic diameter; LVEF, left ventricular ejection fraction; CRRT, continuous renal replacement therapy; ECMO, extracorporeal membrane oxygenation; VT/VF, ventricular tachycardia/ventricular fibrillation.

Next, we evaluated the treatments and clinical complications in both groups. Patients in the high-ACEF group had higher rates of prescriptions for diuretics, dopamine, and norepinephrine. They also had a greater need for inotropic agents, ventilator supports, IABP, CRRT, and ECMO than those in the low-ACEF group. This implied that the patients in the high-ACEF group had more serious conditions. By contrast, no significant differences were observed between the two groups with respect to treatment with renin-angiotensin system inhibitors, beta-receptor blockers, aldosterone antagonists, vitamin C, immunoglobulins, methylprednisolone, and temporary use of pacemakers. These results demonstrated that the patients in the high-ACEF group needed more medical support and were in worse condition than the patients in the low-ACEF group (Table 3).

The patients with fulminant myocarditis in the high-ACEF group were more likely to develop clinical complications [shock, NYHA III-IV, ventricular tachycardia/ventricular fibrillation (VT/VF), multiple organ failure, and death] than the patients in the low-ACEF group. This indicated that patients in the high-ACEF group were at greater risk of serious adverse cardiac events. Importantly, the mortality rate of patients with acute fulminant myocarditis was 38.0% in the high-ACEF group and 2.94% in the low-ACEF group (Table 3).

### Evaluation of Electrocardiographic Data at 30 Days and the Cumulative Rates of MACE at 1 Year in Patients With Acute Fulminant Myocarditis

The echocardiographic measurements 1 month after discharge in survivors were compared according to their ACEF scores. Patients in the high-ACEF group had markedly higher LAd [39.70 ± 6.34 vs. 35.24 ± 5.10 mm, *p* < 0.05], LVESd [36.30 ± 6.45 vs. 32.00 (30.00∼35.00) mm, *p* < 0.05)], and LVEDd [51.53 ± 5.24 vs. 49.00 (45.00∼52.00) mm, *p* < 0.05)], but remarkably lower LVEF [0.55 ± 0.98 vs. 0.62 (0.58∼0.68), *p* < 0.05]. These data also indicated greater prevalence of weakening motion of the ventricular wall and valve regurgitation in the high-ACEF group. These results showed that high ACEF scores were closely correlated with myocardial recovery at 1 month in patients with acute fulminant myocarditis (Table 4).

**TABLE 4:** Echocardiographic data at 30 day in patients with acute fulminant myocarditis.

Echocardiographic data	ACEF score ≤ 1.43 (*n* = 165)	ACEF score > 1.43 (*n* = 31)	*P*-value
LAd (mm)	35.24 ± 5.10	39.70 ± 6.34	0.088
LVESd (mm)	32.00 (30.00∼35.00)	36.30 ± 6.45*	0.001
LVEDd (mm)	49.00 (45.00∼52.00)	51.53 ± 5.24*	0.01
LVEF	0.62 (0.58∼0.68)	0.55 ± 0.98*	0.001
Pericardial effusion [*n* (%)]	33 (20.00%)	6 (19.35%)	0.934
Weakening motion of the ventricular wall [*n* (%)]	44 (26.67%)	22 (70.97%)*	0.000
Valve regurgitation [*n* (%)]	42 (25.45%)	19 (61.29%)*	0.000

Values are presented as mean ± standard deviation, median and interquartile range or number and percentages. *P < 0.05 (ACEF score > 1.43 vs. ACEF score ≤ 1.43). ACEF, the age, creatinine, and left ventricular ejection fraction; LAd, left atrium diameter; LVESd, left ventricular end-systolic diameter; LVEDd, left ventricular end-diastolic diameter; LVEF, left ventricular ejection fraction.

**FIGURE 2:**
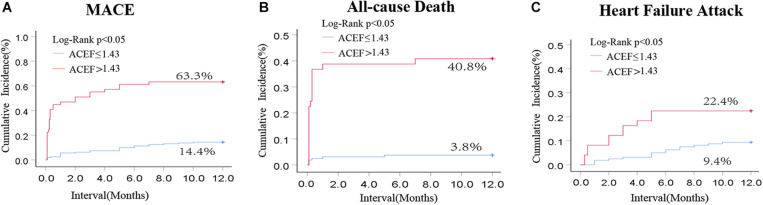
Kaplan-Meier curve for cumulative rates of MACE **(A)**, all-cause rate **(B)**, and heart failure attack **(C)** according to different levels of the ACEF score in patients with cute fulminant myocarditis.

Those patients were followed up for 1 year. Among them, 160 patients in the low-ACEF group (ACEF ≤ 1.43) and 49 patients in the high-ACEF group (ACEF > 1.43) were included while 11 patients were lost in the follow-up period. The rates of MACE, all-cause death, and cardiac failure attack at 1 year were remarkably higher in the high-ACEF group compared to those patients with low ACEF scores (Figure 2). These data clearly demonstrated the value of the ACEF score for predicting 1 month and 1 year outcomes in patients with acute fulminant myocarditis.

## Discussion

This study successfully analyzed the differences in clinical presentation of patients with acute fulminant myocarditis, and it established one simple and precise ACEF score assessment tool. It found that patients with high ACEF scores had more severe disease conditions, required more medical treatments, and possibly had higher clinical complications and mortality rates than the patients with low ACEF scores. In addition, ACEF scores demonstrated a strong ability to predict recovery of cardiac function in 30 day survivors and the risk of MACE, all-cause death and cardiac failure attack in patients with acute fulminant myocarditis. Thus, the ACEF score was shown to be a valuable predictor for patients undergoing acute fulminant myocarditis in terms of assessing their risk of in-hospital mortality and long-term prognosis.

A total of 220 patients with acute fulminant myocarditis were included in the present study. The patients with acute fulminant myocarditis in the non-survivor group presented with a broad spectrum of symptoms and severe cardiac dysfunction, and they needed more medical treatments and circulatory support or heart transplantation. Our critical findings were in accordance with previous results (Ammirati et al., 2018; Veronese et al., 2018). Early risk stratification contributed to patients with acute fulminant myocarditis due to high short-term and long-term mortality. In previous studies, many risk factors were found to be related to poor prognosis for developing fulminant myocarditis in patients, especially echocardiographic data and kidney injury (Yang et al., 2012; Xu et al., 2018), and prolonged PR interval and widened QRS complex (Sun et al., 2017). The echocardiographic features of myocarditis in the non-survivor group were often non-specific, but evaluating heart function with echocardiographic data was helpful in determining prognosis. In the current study, the patients in the non-survivor group were older, had higher serum creatinine, and had lower LVEF than the patients in the survivor group. Thus, the predictive ability of a single factor was proven to be insufficient. Among many parameters (heart rate, WBC count, CK-MB, total bilirubin, LVESd, and ACEF), the ACEF score at admission, by incorporating three easily obtainable variables (age, creatinine, and LVEF), was independently associated with an unfavorable prognosis, and it was a predictor of in-hospital mortality in patients with acute fulminant myocarditis.

Early estimation of prognosis in patients with acute fulminant myocarditis is difficult due to limited clinical studies on long-term outcomes (Sharma et al., 2019). This new ACEF score was simpler to establish and more accurate for developing a prognosis for acute fulminant myocarditis. A high ACEF score probably reflected the more serious conditions and worse prognosis of patients with acute fulminant myocarditis. Thus, patients with high ACEF scores may benefit from early invasive management and more aggressive use of hemodynamic support devices. The ACEF score previously was recommended for evaluating mortality risk in cardiac surgery, and it was considered to be an independent predictor for in-hospital and long-term mortality in patients with infective endocarditis (Ranucci et al., 2009; Wei et al., 2019). Moreover, the ACEF score had been used to stratify the risk of 1 year clinical outcome and prognostic impact in 30 day survivors of acute myocardial infarction after percutaneous coronary intervention (Lee et al., 2015; Stähli et al., 2018; Gao et al., 2020). The current study was accomplished by evaluating the predictive ability of the ACEF scores. A higher ACEF score markedly indicated worse clinical course in hospital, a poor recovery of cardiac function at 30 days, and higher rates of MACE and death in patients who suffered from acute fulminant myocarditis. Clinical sepsis produced substantial cardiomyocytes injury which was closely correlated to a reduced peak of intracellular Ca^2+^ sequestration, but no changes in resting intra-cellular Ca^2+^ and Ca^2+^-transient decay. It is possible that fulminant myocarditis leading to low cardiac output syndrome, shock and life-threatening arrhythmia, might be attributed to alterations in Ca^2+^ transient properties and the mechanical properties (Ren et al., 2002). Consistently, this study determined that it was acceptable to use the ACEF score to predict short-term and long-term outcomes in patients after acute fulminant myocarditis.

## Limitations

Some limitations inherent to the study design should be acknowledged. First, the number of patients referred for acute fulminant myocarditis was rather small. Second, the proposed ACEF score risk categories must be tested in an external validation cohort. Third, although a comprehensive group of variables was used in the multivariate models, not all risk scores developed for the multivariate models were included.

## Conclusion

In this study, the ACEF score, which incorporates three objectively measurable risk factors (age, creatinine level, and LVEF), is an extremely simple, practical, easy-to-calculate, and user-friendly tool for determining the prognosis in the acute fulminant myocarditis patient population. Furthermore, in contrast to other risk scores, the ACEF score allows for the identification of risk stratification, adverse events, and prognosis, which may further influence management decisions in acute fulminant myocarditis. These findings strengthened the role of the ACEF score and demonstrated that it had better predictive ability and could independently predict clinical adverse events, in-hospital mortality, cardiac function after 1 month of recovery, and 1 year prognosis in patients presenting with acute fulminant myocarditis. The ACEF score provided a novel and effective indicator to stratify the risk for patients with acute fulminant myocarditis.

## Data Availability Statement

The original contributions presented in the study are included in the article/supplementary material, further inquiries can be directed to the corresponding author/s.

## Ethics Statement

The studies involving human participants were reviewed and approved by the Ethics Committee of First Affiliated Hospital of Soochow University. Written informed consent to participate in this study was provided by the participants’ legal guardian/next of kin. Written informed consent was obtained from the individual(s), and minor(s)’ legal guardian/next of kin, for the publication of any potentially identifiable images or data included in this article.

## Author Contributions

MX and TJ: designing the study. LL, XY, and YG: data collection and analysis. XY, JX, and MX: statistics. MX, LL, and XY: manuscript preparation and writing. MX: English improvement. TJ: funding support. All authors contributed to the article and approved the submitted version.

## Conflict of Interest

The authors declare that the research was conducted in the absence of any commercial or financial relationships that could be construed as a potential conflict of interest.
